# Multi-dimensional discovery of biomarker and phenotype complexes

**DOI:** 10.1186/1471-2105-11-S9-S3

**Published:** 2010-10-28

**Authors:** Philip RO Payne, Kun Huang, Kristin Keen-Circle, Abhisek Kundu, Jie Zhang, Tara B Borlawsky

**Affiliations:** 1 Department of Biomedical Informatics, The Ohio State University, 3190 Graves Hall, 333 West 10th Avenue, 43210, Columbus, Ohio, USA; 2 Center for Clinical and Translational Science, The Ohio State University, Suite 205, 376 West 10th Avenue, 43210, Columbus, Ohio, USA; 3 Comprehensive Cancer Center Biomedical Informatics Shared Resources, The Ohio State University, 3190 Graves Hall, 333 West 10th Avenue, 43210, Columbus, Ohio, USA; 4 Mt. Carmel College of Nursing, 127 South Davis Avenue, 43222, Columbus, OH, USA; 5 Department of Computer Science and Engineering, The Ohio State University, 2015 Neil Avenue, 43210, Columbus, Ohio, USA

## Abstract

**Background:**

Given the rapid growth of translational research and personalized healthcare paradigms, the ability to relate and reason upon networks of bio-molecular and phenotypic variables at various levels of granularity in order to diagnose, stage and plan treatments for disease states is highly desirable. Numerous techniques exist that can be used to develop networks of co-expressed or otherwise related genes and clinical features. Such techniques can also be used to create formalized knowledge collections based upon the information incumbent to ontologies and domain literature. However, reports of integrative approaches that bridge such networks to create systems-level models of disease or wellness are notably lacking in the contemporary literature.

**Results:**

In response to the preceding gap in knowledge and practice, we report upon a prototypical series of experiments that utilize multi-modal approaches to network induction. These experiments are intended to elicit meaningful and significant biomarker-phenotype complexes spanning multiple levels of granularity. This work has been performed in the experimental context of a large-scale clinical and basic science data repository maintained by the National Cancer Institute (NCI) funded Chronic Lymphocytic Leukemia Research Consortium.

**Conclusions:**

Our results indicate that it is computationally tractable to link orthogonal networks of genes, clinical features, and conceptual knowledge to create multi-dimensional models of interrelated biomarkers and phenotypes. Further, our results indicate that such systems-level models contain interrelated bio-molecular and clinical markers capable of supporting hypothesis discovery and testing. Based on such findings, we propose a conceptual model intended to inform the cross-linkage of the results of such methods. This model has as its aim the identification of novel and knowledge-anchored biomarker-phenotype complexes.

## Background

Translational research is a complex and information-intensive endeavour, involving numerous actors, tools, workflows and data types. Several recent reports have defined the ***translational research cycle*** as the bidirectional translation of knowledge and evidence between the “bench” and the “bedside”. Within this translational cycle, Sung and colleagues [1] have defined two critical blockages that exist between basic science discovery and the design of prospective clinical studies (i.e., T1 block), and subsequently between the knowledge generated during clinical studies and the provision of evidence-based care in the clinical or public health settings (i.e., T2 block). A commonly cited phenomenon contributing to the aforementioned T1 block is the lack of tools capable of enabling the systematic discovery of meaningful relationships between biomarkers and phenotypes [2]. This issue is particularly pressing given the dimensionality and scale of information generated by modern sources such as Electronic Health Records (EHRs) and laboratory instrumentation (e.g., genomic and proteomic expression profiling). Unfortunately, generalizable approaches for creating integrative, multi-dimensional networks spanning a range of granularities from bio-molecules to clinical phenotypes are largely absent in the current literature, with the exception of a few notable cases [3,4]. It is this gap in knowledge and practice that motivates the studies and resultant models that we will report upon in this article.

Given the preceding motivation, in the following sub-sections we will: 1) review the experimental context for our work; and 2) introduce three methodological approaches used in our study to induce and reason upon multi-dimensional networks of genes, clinical features, and conceptual knowledge:

### Experimental context

The experimental context for our work stems from a collaboration with the NCI-funded Chronic Lymphocytic Leukemia Research Consortium (CLL-RC, cll.ucsd.edu). The CLL-RC coordinates and facilitates basic and clinical research on the genetic, biochemical and immunologic bases of Chronic Lymphocytic Leukemia (CLL), which is the most common adult leukemia in the United States [5]. The CLL-RC specifically focuses on the identification of phenotypic ↔ bio-molecular relationships that may improve clinical diagnosis/staging and assist in designing personalized treatment plans and evaluating resultant patient responses. The consortium utilizes a shared data management platform, known as the CLL-RC Integrated Information Management System (CIMS), in order to collect, store and analyze longitudinal bio-molecular and clinical data. Such data sets are derived from a cohort of over 5,000 patients spanning a time frame of up to eleven years at the time of this submission. Of note relative to this experimental context and the methods and results described in this report, we have utilized three well-known markers for CLL diagnosis and staging to verify and validate our findings. Specifically, the markers ZAP70 (zeta-chain associated protein kinase 70kDa) and CD38 (cluster of differentiation 38) are used, given their previously demonstrated ability to predict IgVH (immunoglobulin heavy chain variable region) mutational status, a clinical phenotype commonly associated with differentiating between diagnoses of aggressive versus indolent CLL [5].

### Gene co-expression network induction

Gene co-expression networks are established by linking genes with similar expression profiles in data sets derived from subjects that demonstrate a shared clinical phenotype of interest. The similarity of such expression profiles are often measured by parameters such as the Pearson Correlation Coefficient (ρ, -1 ≤ ρ ≤ 1), with ρ = 1 implying perfect correlation and ρ = -1 being a perfect negative correlation [6]. Analyses of such similarities in gene expression are usually employed to identify networks of genes that may serve to predict disease prognosis or treatment outcomes [7].

### Clinical feature co-expression network induction

Similar to the induction of gene co-expression networks, clinical co-expression network induction involves the discovery and quantification of significant motifs of clinical attributes in large-scale data sets [3]. The general methodological pattern for such approaches involves a multi-step process of:

• Selecting a set of potentially interesting clinical variables within a large-scale data set, and normalizing or “binning” their values;

• Aggregating variables into a composite data table;

• Calculating correlation ranked parameters (e.g., Spearman’s rank correlation coefficient) spanning all potential variable pairings within the data set;

• Designating thresholds for correlation metrics to identify potentially significant correlation motifs;

• Visualization and expert validation of the resulting clinical feature co-expression networks [3].

These methods are intended to facilitate the identification of clinical features that exhibit similar co-occurrence motifs within multi-dimensional data sets, thus providing the impetus for the retrospective and/or prospective analysis of such patterns and their statistical significance. Of note, we represent such clinical features as a network construct in order to provide for a computationally tractable representation. Such a structure can incorporate both atomic features and the probabilistic relationships that serve to link such features together. This approach has been described frequently in the published literature [8,9].

### Knowledge discovery in databases

**Figure 1:**
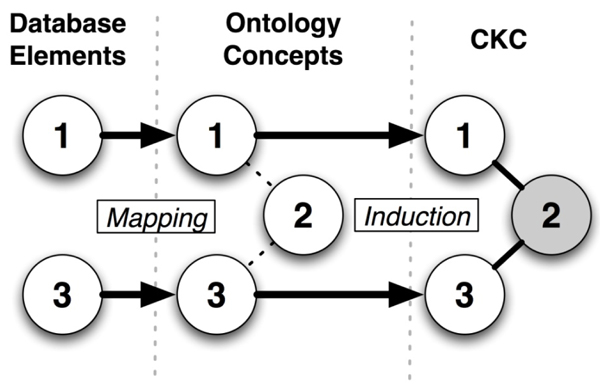
Overview of constructive induction (CI) methodology. Note that Concept 2, which is included in the ontology but does not map to the initial database construct, is used as an *intermediate concept* to define a triplet known as a conceptual knowledge construct (CKC).

Conceptual knowledge can be defined as a combination of atomic units of information *and* the meaningful relationships among those units [10]. The work described in this report utilizes a conceptual knowledge acquisition approach known as *conceptual knowledge discovery in databases* (CKDD). Such CKDD methods focus on the utilization of automated or semi-automated computational methods to derive knowledge from the contents of databases [10]. The use of domain-specific knowledge collections, such as ontologies, is necessary to inform this knowledge induction process since commonly used database modelling approaches do not always incorporate semantic knowledge corresponding to its contents. This overall approach is the basis for a specific CKDD methodology known as *constructive induction (CI)*, as is illustrated in Figure 1. The network-constructs that serve to link together variable of interests using CI are referred to in the remainder of this report as Conceptual Knowledge Constructs (CKCs).

## Results

**Figure 2:**
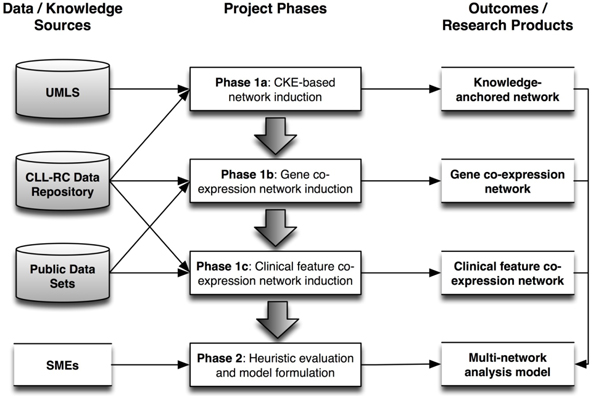
Overview of study phases, data/knowledge sources, and outcomes/research products.

In the following sub-sections, we will present the results generated during the course of our multi-methodology study. These results are organized per the phases of our overall research design, and their interrelationships are explicitly represented in the illustration of our study design provided in Figure 2.

### Phase 1a: CKE-based network induction

A corpus of 107 data elements was extracted from the CIMS data dictionary, of which 68 (63.5%) and 39 (36.4%) corresponded to phenotypic and bio-molecular variable types, respectively. Those data elements were then mapped to concepts found in SNOMED-CT (Systematized Nomenclature of Medicine - Clinical Terms) and the NCI Thesaurus ontologies, using the UMLS (Unified Medical Language System) Knowledge Source Server lexical search tool. The initial 107 data elements mapped to 882 (537 unique) ontology concepts, of which 455 (51.6%) and 427 (48.4%) corresponded to the initial phenotypic and bio-molecular concepts respectively. Using the previously described CI method, 5800 CKCs were generated. Five subject matter experts (SMEs) with expertise in the CLL domain evaluated the validity (defined as the factual accuracy of the CKC) and meaningfulness (defined as the ability of the CKC to inform a novel hypothesis) of a random sample of 66 CKCs, indicating that 24.2%, 65.2%, and 10.6% were completely valid, partially valid/invalid, and completely invalid, respectively. The SMEs further evaluated those CKCs designated as completely valid, and concluded that 90% were meaningful and could be used to formulate a novel hypothesis for further testing.

### Phase 1b: Gene co-expression network induction

The GDS1388 and GDS1454 data sets were retrieved from the Gene Expression Omnibus (GEO) database, comprising 12,651 probe sets. The results for correlation (Pearson Correlation Coefficient**, ρ)** and intersection analysis as applied to these data sets are summarized in Table 1. For ZAP70, 38 genes were present in both gene lists, and similarly, 8 genes were obtained for CD38. Using the CODENSE (Coherent Dense Subgraphs) algorithm [11], we identified 45 highly connected co-expression networks. Using iterative analyses, a particularly promising network with 51 genes, including ZAP70 and CD38 was identified. For the genes in this co-expression network, we compared their expression levels between the 49 patients without IgVH mutation and the 51 patients with IgVH mutations as found in the GDS1454 data set. Of note and as introduced earlier, IgVH mutation status is one of a small number of well-characterized and prospectively validated biomarkers for predicting CLL disease progression [5]. The genes with significant differences between the two groups were further tested for their capacity of predicting IgVH mutation status using a supervised linear classifier and a cross validation with 20% sample holdout. These analyses indicated that the genes that share the same co-expression network with ZAP70 identified using the preceding approach are enriched with genes that show significant differential expression between the IgVH unmutated and mutated groups.

**Table 1:** CD38 and ZAP70 gene list intersections. The p-value’s reported in this table are generated using a Fishers exact test.

		GDS #			
		
		1388	1454	Intersection	p-value		
	PCC>0.4 (# of genes )	*639*	*114*	*8*	*0.263*
**CD38**					
	PCC<-0.4 (# of genes)	*269*	*1*	*0*	*1*

	PCC>0.4 (# of genes)	*944*	*55*	*8*	*0.0543*
**ZAP70**					
	PCC<-0.4 (# of genes)	*575*	*124*	*30*	*<1e-6*

### Phase 1c: Clinical feature co-expression network induction

**Figure 3:**
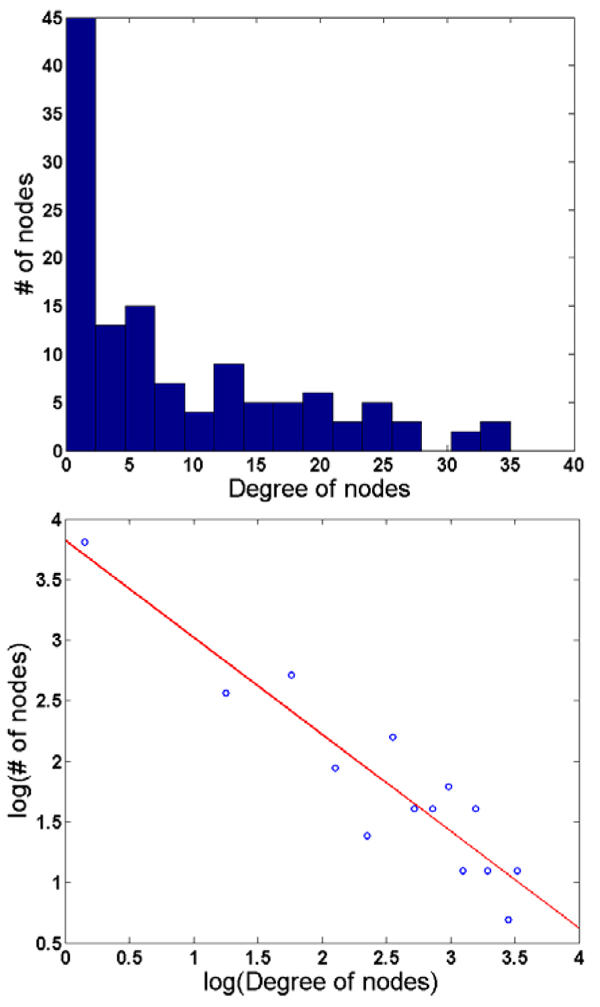
Top: Histogram of the number of nodes in the clinical attribute network created during Phase 1c. Bottom: Using a log-log scale, the histogram can be fitted by a straight line (red, R=0.93).

As is described in our methods, we calculated a 125x125 correlation matrix using a data set corresponding to a random sample of 1516 patient encounters recorded in CIMS (where those encounters corresponded to a longitudinal series of events for a given patient). In order to focus on the highly correlated attributes, we set a threshold of ρ≥ 0.95, and generated a clinical attribute network from those attributes satisfying such criteria. A histogram representing the distribution of such values was created and fitted by a straight line in log-log scale, implying that the induced network is scale-free (Figure 3). We examined the top ten nodes in our network that exhibited the highest degree of connections. Interestingly, seven of them are related to common CLL-related laboratory measurements, including: hemoglobin, billirubin, creatine, albumin, calcium, red blood cell count, and alkaline phosphate. The remaining three nodes are all prognostic markers, including: IgVH homology and ZAP70 gene expression. When this network was visualized using Pajek (http://pajek.imfm.si/doku.php), and examined by an SME, it was determined that within the core network, the majority of the attributes corresponded to clinical phenotypes, cytogenetics, and participant demographics. In contrast, at the boundary of the core network, the majority of variables corresponded to prognostic markers that served to interface core nodes with the peripheral nodes. Another interesting observation is that all but one of the attributes related to quantitative immunophenotyping measurements were in the peripheral areas of the visualization.

### Phase 2: Heuristic evaluation and model formulation

**Figure 4:**
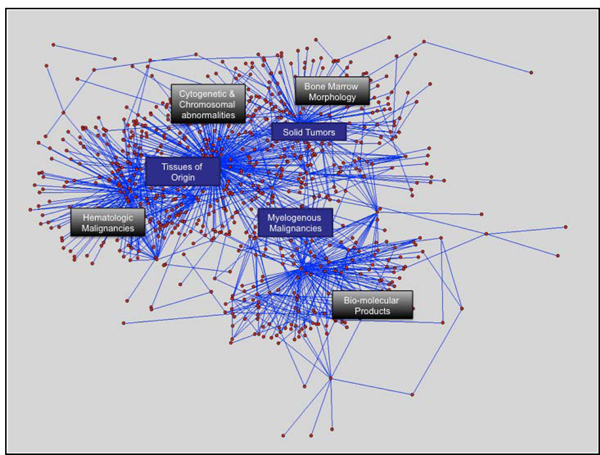
Energy-minimized graph visualization of semantically anchored union of network constructs generated in Phases 1a-1c, with significant groups of nodes annotated to indicated broad concept classes.

An SME with over ten years of CLL-related research experience reviewed and annotated the possible interrelationships between the networks generated in Phases 1a-c using the methods previously described, notably including an inspection of an energy-minimized graph visualization of the composite network created by the semantically-anchored union of the three preceding networks (Figure 4). This analysis indicated the following two characteristics:

1) Of the variables included in the random sample of CKCs from phase 1a that were considered both valid and “meaningful”, there was 100% overlap between the initial or terminal concepts included in those CKCs and the concepts included in the top ten clinical nodes identified in phase 1c; and

2) For each gene identified as part of the same CKCs identified as being novel and “meaningful”, there was at least one or more linkages between those genes and analogous genes included in the co-expression network generated during phase 1b.

Based upon these findings, our SME hypothesized that multi-dimensional and clinically relevant complexes of biomarkers and clinical phenotypes could be generated through the valid, semantic linkages spanning the three networks. An exemplary instance of such a “vertical linkage” is shown in Table 2.

**Table 2:** Exemplary intersection between gene co-expression, knowledge-anchored, and clinical feature co-expression networks, as identified during Phase 2.

Network	Initial Concept	Network Path or CKC	Terminal Concept
Gene Co-Expression*	CD8A	CD8A↔IL2RB↔ZAP70	ZAP70

Conceptual Knowledge**	ZAP70	[ZAP70 gene]-*gene_plays_role_in_process*-[Ligand Binding]-*biological_process_involves_gene_product*-[LTB4R protein, human]-*gene_product_expressed_in_tissue*-[Lymphoid Tissue]-*is_normal_tissue_origin_of_diseas*e-[Chronic lymphocytic leukaemia refractory]	Chronic lymphocytic leukemia refractory

Clinical Feature Co-Expression*	Chronic lymphocytic leukemia refractory	Chronic lymphocytic leukemia refractory (treatment response) ↔ del(17p13) ↔ Chronic Lymphocytic Leukemia with Unmutated Immunoglobulin Heavy Chain Variable-Region Gene ↔ Lactic acid dehydrogenase raised	Lactic acid dehydrogenase raised

* Network path; ** CKC (including semantic relationships in *italics*)

## Discussion

**Figure 5:**
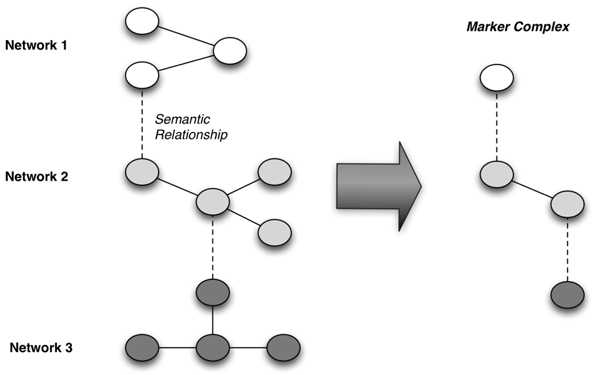
Illustration of heuristically derived conceptual model for multi-dimensional marker complex induction and aggregation.

As was stated at the outset of this report, the ability to identify and reason upon complexes of biomarkers and clinical phenotypes is prototypical of the information needs incumbent to both translational research and personalized healthcare. In this report, we have described the application of three different methods for addressing such needs, spanning levels of granularity from bio-molecules to domain knowledge sources, all within a shared experimental context. We have also presented preliminary findings, and a heuristically derived model, for the “vertical” integration of such networks. Such an integrated network can ultimately be employed to identify and evaluate higher-order bio-marker-to-phenotype systems that may have basic science and/or clinical significance (Figure 5). However, despite these promising results, a number of critical limitations should be noted, including: 1) the demonstration of our methods and findings relative to a single, disease-focused data set; 2) the continued absence of automated methods to instantiate “vertical” linkages, spanning bio-marker and clinical phenotype networks at various levels of granularity; and 3) the limited number of SMEs engaged in the validation of our initial results. Of note, relative to the first of these limitations, is the non-domain specific nature of the computational methods employed in our studies. We believe that such methods can be readily applied to a broad variety of disease areas, such as current efforts by the authors to verify and validate our approach to multi-network analyses in a number of immunologic and musculoskeletal diseases. In light of the remaining limitations, as part of future efforts in this area of research, we plan to: 1) implement and evaluate a semi-automated knowledge-anchored “vertical” network integration pipeline; and 2) engage a broader audience of SMEs in systems-level evaluations of our results.

## Conclusions

Given the results described in this report, we believe that our initial findings provide the basis for a new, multi-dimensional approach to the discovery of knowledge-anchored biomarker and clinical phenotype complexes. These complexes have the potential to increase our understanding of critical disease domains at a comprehensive and systems level. Ultimately, the derivation of these types of systems-level models on a regular basis is critical to realizing the anticipated benefits of translational research and personalized medicine.

## Methods

The objective of our study was to answer the following three research questions relative to the results generated via the use of the three preceding methods as applied to the CIMS data sets:

• *Can the results of such methods be combined into an aggregate construct?*

• *Does such a composite construct provide for the discovery of valid and potentially novel networks in large-scale translational data sets?*

• *Does such a composite construct include higher-order complexes of markers spanning a spectrum of granularity from bio-molecules to clinical phenotypes?*

In order to address these questions, we employed a four-phase approach as illustrated in Figure 2 and described below. During this process, we both verified and validated the data sets and methods employed during the course of several preliminary studies [12-14], and developed novel methods to integrate and analyze the results of such approaches:

### Phase 1a: CKE-based network induction

In the first phase of our study, a set of data elements from the CIMS data dictionary were selected by two SMEs and utilized for CI analyses, which were implemented using a set of specialized PERL scripts and leveraged the contents of the UMLS. During this process, a novel graph-theoretic technique was devised, controlling the CI process to ensure that only concepts with similar levels of semantic “granularity” were included in resulting CKCs. A random sub-set of these CKCs was selected, and evaluated by five SMEs to determine both validity and meaningfulness (e.g., the ability to inform novel hypotheses). The resulting valid and meaningful CKCs were aggregated as a graph constructs, using ontology-anchored relationships between constituent elements to form a network.

### Phase 1b: Gene co-expression network induction

In the second phase of our study, the GEO database was queried using the phrase "chronic lymphocytic leukemia". Of the returned data sets, those that did not include overlapping patient cohorts were selected for further analysis. Of note, these data sets had already been normalized, per the conventions imposed by the GEO repository. Using a MATLAB script and the well-characterized ZAP70 [5] gene as an “anchor”, **ρ** values were calculated for all possible gene pairs in the preceding data sets. The resulting output file was comprised of a list containing only those genes found with |**ρ**| ≥ 0.4 or higher, a threshold selected based upon current best practices for such co-expression analyses. For comparison purposes, we carried out the same operations using the similarly well-characterized CD38 marker [5] as an “anchor”. In both instance, the significance of such co-expression was measured using a Fishers exact text. To determine which gene symbols could be found in multiple correlation gene lists, an intersection analysis was performed using a MATLAB script. Building upon the results generated using these methods, we again queried GEO using the phrase "metastatic cancer”, and selected datasets containing both normal and tumor tissues obtained from a primary biopsy, resulting in 19 datasets for different cancers. The **ρ** values for every pair of genes in every dataset were calculated, and filtered using a threshold set at **|ρ|** ≥ 0.75. Subsequently, the CODENSE algorithm [15] was applied to this data set in order to construct a network where each edge linking a pair of genes appeared in at least 4 component datasets. The network motifs that had a connectivity ratio *r* > 0.4 (i.e., given a co-expression network with *K* nodes and *L* edges, *r = L*/(*n*(*n-1*)/*2*)) were selected for further analyses [11]. The networks that contained ZAP70 and CD38 were selected for further analyses in the context of their ability to predict IgVH mutational status, a clinical phenotype commonly associated with differentiating between diagnoses of aggressive versus indolent CLL, as was noted earlier in our report [5].

### Phase 1c: Clinical feature co-expression network induction

A set of 182 clinical variables from the CIMS repository were selected based upon SME input, and the values associated with the variables were aggregated into a single data table based upon shared temporal relationships (e.g., each set of variables corresponded to a single research participant encounter). Each categorical variable was binned per the given categorical responses. In cases where more than eight individual categories were present, bins were created for ranges of categories. To further assist in defining parameters for the data sets, continuous clinical and laboratory variables were compared to the Ohio State University Medical Center's (OSUMC) laboratory and clinical data reference ranges. If the data field corresponded to an existing OSUMC reference range, it was used to create bins for below normal, normal, and above normal. Due to the categorization of the data set via the previously described binning process, we used the *Spearman rank* correlation method to calculate the correlation coefficient between pairs of attributes. We ignored null entries and considered only non-null pairs of entries in order to find the correlation between attributes. This analysis yielded a 125x125 correlation matrix. Due to null values and incomplete entries, there were some cases where only a few data points (as low as 2) were available to calculate a correlation. Since this phenomenon could potentially produce statistically unreliable correlation values, we set a threshold of 20 as the minimum number of non-null data points needed to reliably calculate the correlation. If the number of non-null data points was less than 20, we designated in our results that no correlation between that pair of attributes existed. The resulting attribute network was visualized using the Pajek software package, with a layout mode selected that embedded the nodes in a 2D plane with maximal energy preserved.

### Phase 2: Heuristic evaluation and model formulation

In our final study phase, we compared and integrated the findings generated in Phases 1a-c. Specifically, we executed a breadth-first search of all possible pair-wise linkages between the network generated in Phase 1a and those generated in Phases 1b-c, using the contents of the UMLS semantic network to identify knowledge-anchored relationships between vertex pairs (implemented as a JAVA application, leveraging an RDF [Resource Description Framework] representation of the networks). This approach employs both the parent-child and sibling relationships found in the UMLS to enable the identifications of high-order relationships between the networks. Subsequently, an SME reviewed and annotated the resulting pair-wise relationships based upon their perceived validity. A multi-network model, made up of valid cross-linkages, was then induced and visualized using the open-source Graphviz (http://www.graphviz.org) software package. Such a model is intended to aid in the identification of marker complexes spanning multiple levels of granularity, and derived from differential methods and knowledge sources.

## Authors' contributions

PROP, KH and TBB conceived of the study, oversaw its design and execution, analyzed the aggregate study results, and drafted this article. KKC, AK and JZ participated in the design of the study, the execution of phases 1a-1b, the analysis of the resulting data sets, and participated in the preparation of this article. PROP executed phase 2 and analyzed its results.

## Competing interests

The authors declare that they have no competing interests.
